# Towards High Capacitive Performance of Chemically Deposited β-Ni(OH)_2_ Nanolamellae Electrode Films

**DOI:** 10.3390/mi14081644

**Published:** 2023-08-20

**Authors:** Kevin Radakishna Moonooswamy, Mohammed Es-Souni

**Affiliations:** 1 Currently with Toronto Metropolitan University, Victoria Street 350, Toronto, ON M5B, Canada; kevin.moonoosawmy@torontomu.ca; 2 Formerly with Kiel University of Applied Sciences, Grenzstrasse 3, 24149 Kiel, Germany

**Keywords:** nanolamellae, nickel hydroxide, chemical bath deposition, supercapacitors, electrochemical energy storage, surface modification

## Abstract

Nickel hydroxide β-Ni(OH)_2_ nanolamellae with high aspect ratios were grown via chemical bath deposition (CBD) on both smooth and textured nickel foil. Depending on bath composition and/or the presence of an additive, thin foam-like nanolamellae to stacked lamellae were obtained. The used CBD method is highly cost-effective, as it is faster and requires less chemicals than typical hydrothermal methods, and it is readily implementable for large-scale production. The influence of surface texture on the final morphology and its effect on capacitive performance was investigated. Herein, we show how subtle changes in the concentration can drastically influence the morphology, which, in turn, drastically impacts the supercapacitive performance of the electrode. Also, the use of a textured surface significantly impacts the morphology, with vastly better cycling performance than samples made on a relatively smooth substrate. The measured specific capacitance values of the best sample were 1961 Fg^−1^ at 5 mVs^−1^ and 1998 Fg^−1^ at 1 Ag^−1^ under potentiostatic and galvanostatic conditions, respectively. This sample also retained 100% of its initial specific capacitance when discharged at a very high current density of 40 Ag^−1^. These values are substantially enhanced compared to previously reported data using a nearly analogous method (CBD with higher reagent conc.), with our method, cost-wise, offering economic advantages relative to results obtained with similar materials and other methods (e.g., hydrothermal).

## 1. Introduction

Nickel hydroxide is an important component as an electrode material in energy storage devices such as batteries and supercapacitors. Supercapacitors based on pseudocapacitance derive their capacitive properties from changes in oxidation state during a redox reaction, where the generated charge is proportional to the applied potential difference. The presence of multiple oxidation states is a salient feature of transition metals. Their oxides—or hydroxides in certain cases—are not only often chosen based on their capacitive properties but also their combined chemical and physical properties, such as conductivity, corrosion resistance, thermal stability, and low-cost processability, together with morphology control, which, in turn, influences surface area/porosity. Ni is an earth-abundant material whose oxides and hydroxides have high chemical and thermal stability. They are an environmentally friendly materials and relatively cheap when compared to the prohibitive cost of RuO_2_, despite the latter displaying better capacitive performances [1]. Ni(OH)_2_ has garnered some attention due to its high theoretical capacitance of 2082 Fg^−1^ within a 0.5 V potential window [2]. One of the highest specific capacitance values reported for Ni(OH)_2_ on Ni foam is 3152 Fg^−1^ [3], although this value drops by ~48% after 300 cycles. The foam is made by coating reticulated polymer foam with Ni metal using either electroplating or CVD, which adds to the foreseeable cost of manufacture [4]. This highlights the need to further improve the cycling stability of such systems while reasonably maintaining or enhancing capacity retention.

Current synthetic methods used to produce Ni(OH)_2_ are generally encompassed by techniques such as electrochemical deposition/templating [3,5,6], sol–gel processing [7,8], hydrothermal techniques [9,10,11,12,13], and chemical bath deposition (CBD) [14,15,16]. Electrochemical deposition often yields α-Ni(OH)_2_, with specific capacitance in the range of 578 Fg^−1^ to 3152 Fg^−1^ [3,5], with the latter having poor cycling performance due to adhesion problems [3]. The sol–gel method is used to generate Ni(OH)_2_ but mostly as a precursor for NiO formation, as an annealing step is required to calcine the film [7,8]. The range of specific capacitance obtained from hydrothermal and CBD are 324 [13] to 1778 [12] Fg^−1^ and 398 [14] to 1098 [15] Fg^−1^, respectively. However, the hydrothermal method requires the use of an autoclave, temperatures near or above 100 °C, and synthesis time in the order of tens of hours. For example, one of the highest specific capacitances reported for β-Ni(OH)_2_ using the hydrothermal method required 24 h autoclaving at a temperature of 120 °C [12], which inevitably leads to an increase in terms of both financial cost and carbon footprint for this method. Moreover, a binder is necessary to anchor the crystals onto a substrate for electrochemical characterization, which decreases the extent of the available active surface area. The CBD method provides a low-cost alternative, resulting in crystals grown directly on a substrate over a relatively shorter period of time. A major advantage of the CBD method over the hydrothermal method lies in its suitability for large-area processing and upscalability, which positively counterbalances its drawbacks, such as limited control for epitaxial growth (thin films <30 nm [17], as defined by the height of film with respect to the plane of the substrate) and chemical waste; the latter can be mitigated via recycling protocols.

Nevertheless, both the hydrothermal and CBD methods provide a major platform to a wide variety of accessible (meso, micro, and nano) morphologies, such as flakes [9], ribbons [10], lamellae [11], tubes [18], rosettes [11], flower-like morphologies [19], spheres [20], sheets [21], and belts [13]. These morphologies, in turn, significantly influence the capacitive performance. It has been suggested that smaller crystallite size [10] and ultrathin nanoscale [22] features, which lead to higher surface area and pore size tailoring [23], have the potential to improve access for the electrolyte, thereby enhancing capacitive performance. Out of the two (α and β) polymorphic forms of nickel hydroxide, the brucite-like hexagonal β structure is generally obtained when using bases such as ammonium hydroxide, KOH, and NaOH. Other additives and combinations thereof have been extensively used to understand their influence on the final morphology. Amines such as ethylenediamine and hexamethylenetetramine (HMTA) have been used to generate lamellae or flakes; chelating agents such as dimethylglyoxime produce tubular structures. Buscaglia et al. [11] systematically investigated the formation of numerous morphologies under the influence of organic additives including polymers, surfactants, and cellulose. However, very little attention has been paid to the influence and/or effect of textured surfaces of a substrate on the resultant morphology of β-Ni(OH)_2_.

As mentioned above, the electrochemical performance of Ni(OH)_2_ very much depends on the morphology of the nanostructure, as well as on substrate/substrate–heterostructure, regardless of the crystal variant (α- or β-Ni(OH)_2_). Most promising are Ni-based substrates, where NiOH_2_ can grow binder-free and ready-to-use. For instance, Li et al. reported on the hydrothermal synthesis of β-Ni(OH)_2_ on Ni foam with a maximum pseudocapacitance of 1470 F/g at 2 A/g and a relatively high retention rate [24]. Carbon nanomaterials, together with modified Ni(OH)_2_, can also be advantageous to boost performance, as described by Xin et al. [25], who synthesized BO_2_^−^-intercalated α-Ni_x_Co_(1−x)_(OH)_2_ on reduced graphene oxide in a hydrothermal process and obtained pseudocapacitance values of up to 2170 F/g, albeit with a relatively moderate retention rate. In another process, Chen et al. [26] reported on the fabrication of β-Ni(OH)_2_ flakes via alkaline etching of NiAl-double-layered hydroxide. The samples were mixed with acetylene black and PVDF and subsequently pasted on Ni foam for characterization. The pseudocapacitance values and the retention rate were moderate, with maximum values of 850 F/g and 50%, respectively. To enhance the conductivity of hydrothermally synthesized Ni(OH)_2_ lamellae, Kim et al. [27] deposited colloidal Au-nanoparticles on them. The samples for electrochemical characterization were prepared using acetylene black, PVDF, and Ni foam, similarly to [26]. The higher conductivity imparted to Ni(OH)_2_ via Au-NPs entailed a maximum pseudocapacitance of 1979 F/g, compared to 1325 F/g for pristine samples. From the above, it appears that Ni(OH)_2_ is mostly synthesized via hydrothermal methods, and in the majority of cases, sample preparation for electrochemical characterization involves mixing the active material with a binder (PVDF) and carbon black before pasting on a metal electrode, preferably Ni foam. Herein, we take advantage of the CBD method to generate different morphologies of β-Ni(OH)_2_ lamellae via specific tailoring of the chemical bath composition. The nanostructures are grown binder-free directly on both smooth and textured nickel substrates, which serve as back contacts. This method simplifies both the synthesis procedure and the ensuing device characterization, rendering both cost-effective and easy to handle. Furthermore, effects of the different morphologies obtained as a result of both the substrate surface texture and additives on the pseudocapacitance performance are systematically investigated to help define optimum process windows for the targeted functionality. Moreover, the method incorporates the advantages of a potentially large-scale implementation, while limiting the inherent downsides of the hydrothermal method. Herein, we show that the current method can lead to high capacitive performance, together with high retention rates, while pursuing a cost-effective procedure driven by our efforts to reduce our carbon footprint.

## 2. Materials and Methods

### 2.1. Chemicals

Nickel chloride hexahydrate (NiCl_2_.6H_2_O), hexamethylenetetramine (HTMA), and polyethyleneimine (PEI), were purchased from Sigma-Aldrich, Darmstadt, Germany. Acetone and isopropanol-99.9% (IsPOH) were acquired from Walter CMP Gmbh (Kiel, Germany). Additionally, 25% ammonium hydroxide (NH_4_OH) and potassium hydroxide (KOH) were supplied by Carl Roth Gmbh (Karlsruhe, Germany). All chemicals were of analytical-grade purity. All solutions were prepared with deionized water (≥18 MΩ/cm).

### 2.2. Sample Preparation

Nickel foil (99.98%, 0.075 mm thick) substrates purchased from Goodfellow Cambridge Ltd. (Huntingdon, UK) were cut to about (1.5 × 3) cm^2^ and sequentially sonicated for 10 min in acetone, IsPOH, and water. The inclined substrate (working surface facing bottom of beaker) was immersed in a beaker containing the growth solution. NiCl_2_·6H_2_O (0.18 g, 40 mM, pH = 3.60) and HMTA (0.14 g, 50 mM, pH = 6.72) were dissolved by stirring (5 min.) in 20 mL of water, followed by sequential and drop-wise addition of PEI (75 µL, pH = 6.80), and 25% NH_4_OH (6 mL, pH = 12.25), which were further stirred for 10 min. The sample prepared using this method was labelled sample 1; sample 2 contained nearly the same reagents, although with less 25% NH_4_OH content (0.6 mL). Sample 3 was prepared in the presence of only NiCl_2_·6H_2_O (0.18 g, 40 mM, pH = 3.60 and 25% NH_4_OH (6 mL, pH = 12.25)). In a typical experiment, a parafilm-covered flask was placed in an oil bath for 2 h that was preheated to 96 °C such that the chemical bath was maintained at 85–88 °C. Subsequently, the nanolamellae anchored on the substrate were thoroughly rinsed with DI water and blown dry in air. The nickel spikes were galvanically electrodeposited on cleaned nickel foil according to a literature report [28]. Briefly, the Ni foil was anodized in 1 M KOH at 1 A for 30 s, then dipped in 20 wt% H_2_SO_4_ and rinsed excessively with water. A nickel electrolyte containing NiCl_2_·6H_2_O (0.8 M), NH_4_Cl (3.7 M), and boric acid (1 M) as pH buffer kept at 60 °C was used to deposit the nickel spikes at 2 Adm^−2^ for 2 min. Only the front side of the sample was coated, as both edges and the back side were masked. The textured Ni substrate was then briefly dipped in 20 wt% H_2_SO_4_, neutralized with 25% NH_4_OH for 5 min, and dried in air to render it more hydrophilic. The textured surface of the substrate was then subjected to the three aforementioned growth conditions (similar to samples 1, 2, and 3), generating new morphologies labelled samples 4, 5, and 6. Table 1 summarizes the reagent compositions and preparation conditions for all the samples. Samples 1, 2, 3, 4, 5, and 6 had mass differences of 95, 68, 811, 59, 138, and 454 µg/cm^2^, respectively. The mass of each sample was computed based on the mass difference between the as-prepared film and the initial weight of the substrate before growth.

### 2.3. Characterization Methods

The samples were investigated by X-ray diffraction (XRD) (X’Pert Pro, PANalytical, Almelo, The Netherlands) in grazing incident geometry with a fixed angle of 1.5°, a step size 0.05° using monochromatic Cu Kα radiation (λ = 1.5418 Å), and a scanning range (2θ) of 10–90°. A Bruker Raman microscope (532 nm laser diode) was used to acquire spectra over a range of 70–3700 cm^−1^, with a spectral resolution of 3–5 cm^−1^, using a backscattering configuration with a 20× objective. Data were collected from numerous spots on the sample and recorded with a fully focused laser power of 20 mW. Each spectrum was accumulated ten times, with an integration time of 15 s. The Raman signal was recorded using a CCD camera. A silicon substrate with a Raman peak position of 520 cm^–1^ was used to calibrate the spectral frequency. The topography of the nanostructured surfaces was characterized using a high-resolution scanning electron microscope (Ultra Plus, ZEISS, Jena, Germany). The electrochemical experiments were performed at room temperature in a standard three-electrode cell using fresh KOH (1 M) as electrolyte, which was degassed (>30 min) prior to each experiment. Cyclic voltammetry (CV) measurements in a range of 0.25 V to 0.75 V were recorded at different scan rates using an electrochemical work station (Zahner, IM6e, Kronach, Germany). Charging–discharging tests and long-term stability tests at different current densities were performed using a source meter (Keithley2400, Keithley, Cleveland, OH, USA). A Pt mesh and Hydroflex probe (Reversible H_2_ reference electrode) were used as counter and reference electrodes, respectively. All potentials are reported with respect to a normal hydrogen electrode (NHE).

## 3. Results and Discussion

### 3.1. Structural Characterization of the as-Prepared Samples

**Figure 1:**
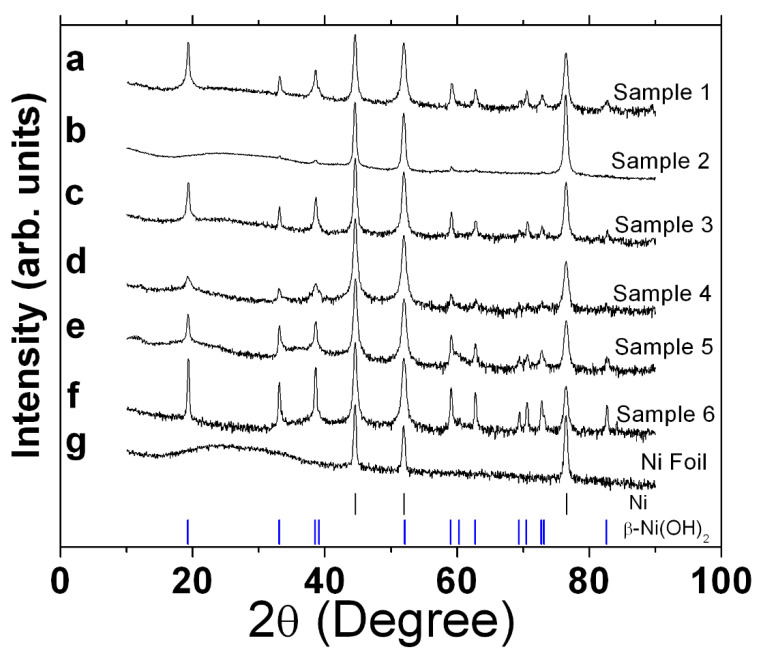
XRD patterns of the as-prepared samples: (**a**) standard procedure β-Ni(OH)_2_ sample 1, (**b**) sample 2 made with lower ammonia content, and (**c**) sample 3 made only in the presence of nickel chloride hexahydrate and ammonia. Samples 4, 5, and 6 shown in (**d**–**f**), respectively, were made using similar procedures as samples 1 to 3 but on a nickel nanospikes surface. The last XRD pattern (**g**) corresponds to the reflexes observed on the bare Ni foil. Vertical bars at the bottom denote the standards for Ni (black) and β-Ni(OH)_2_ (blue).

The XRD patterns of the as-prepared films of samples 1 to 6 are shown in Figure 1a–f, respectively.

The diffraction peaks at 19.3°, 33.2°, 38.6°,59.1°, 62.7°, 70.5°, 72.8°, and 82.6° are attributed to reflections (001), (100), (101), (110), (111), (103), (201), and (202), respectively, of the β-Ni(OH)_2_ phase with a hexagonal brucite [29,30] structure according to PDF 00-014-0117. The XRD patterns for all the samples are similar, with the exception of Figure 1b, where only (100), (101), and (110) are observed at low intensity. The comparatively low-intensity peaks for sample 2 (Figure 1b) qualitatively point towards a sample made up of smaller constituents, implying a relatively thinner sample. Sample 4 (Figure 1d) also shows relatively broader reflexes, whereas sample 5 (Figure 1e) points towards mixed, sharp broad-base reflexes. In contrast, sample 6 (Figure 1f) has well-defined, sharp reflexes. There is a greater level of order within the *ab* plane than along the *c* direction, as the (100) peaks are noted to be sharper than the (001) and (101) counterparts. The average crystallite size of β-Ni(OH)_2_ thin film was calculated on the basis of full width at half maxima intensity of the XRD peak for the (100) plane [30,31] using the Scherrer formula (K = 0.9) [32]. The particle size was found to be approximately 25, 21, 32, 18, 30, and 35 nm for samples 1 to 6, respectively. Figure 1g shows the XRD pattern of the Ni foil, with peaks observed at 44.6°, 52.0°, and 76.6°, which are ascribed to the (111), (200), and (220) reflexes, respectively, consistent with Ni (PDF. 01-070-0989). The X-rays penetrate the entire film, as these Ni peaks are observed in all samples.

**Figure 2:**
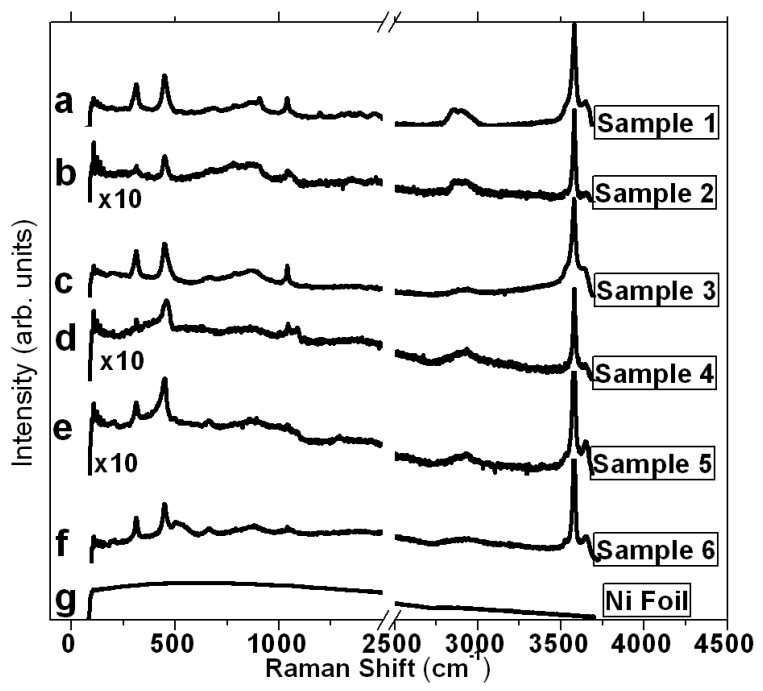
Raman spectra of the as-prepared samples: (**a**) standard-recipe β-Ni(OH)_2_ (sample 1), (**b**) sample 2 made with lower ammonia content, and (**c**) sample 3 made only in the presence of nickel chloride hexahydrate and ammonia. Samples 4, 5, and 6 shown in (**d**–**f**), respectively, were made using similar procedures as samples 1 to 3 but on a nickel nanospikes surface. The Raman spectrum of the bare Ni foil is shown in (**g**). A break in the *x*-axis between 1500 and 2500 cm^−1^, along with a combined offset of the spectra, is warranted to improve clarity.

Further insights can be provided by Raman scattering experiments, which can complement the structural analysis obtained from XRD patterns. β-Ni(OH)_2_ belongs to space group D^3^_3d_ [33]; according to group factor analysis, four active Raman modes (2A_1g_ + 2E_g_) are predicted out of the 12 optical modes [30,34]. Figure 2 shows the spectra of the samples collected on Ni foil; there are no significant differences amongst them, and they correlate well with typical β-Ni(OH)_2_ Raman spectra reported in the literature [30,34]. Four main peaks are observed for all six samples at 313, 449, 868, and 3580 cm^−1^. Despite the presence of these peaks being characteristic of β-Ni(OH)_2_, the assignment of the transitions is debatable [30,34]. Nevertheless, since β-Ni(OH)_2_ has a hexagonal scalenohedral symmetry, which is isostructural to Mg(OH)_2_ (brucite) [29,30], Hall et al. [30] suggested an assignment that parallels that of brucite. The intense peak at 3580 cm^−1^ is associated with the stretching of free hydroxyl [10,35]. The lower Raman intensity observed for sample 2 correlates well with the lower intensity noted from its XRD patterns. This implies less growth of β-Ni(OH)_2_, possibly resulting smaller lamellae for sample 2. The Raman spectra of samples 4 and 5 are also relatively less intense than the other samples but are more resolved than those of sample 2, as further elaborated upon in Section 3.2. Other intermediary peaks are also observed from the Raman spectra and ascribed to second-order modes; the broad peak observed at ~2900 cm^−1^ is correlated with a C-H stretching mode due to surface hydrocarbon, and the sharp and relatively weak peak at 3645 cm^−1^ is also attributed to surface O-H stretching, which is suggested to arise from stacking disorder in β-Ni(OH)_2_ lamellae [30].

### 3.2. Morphological Characterization and Growth Mechanism of the β-Ni(OH)2 Samples

(a) Growth on smooth Ni substrates

**Figure 3:**
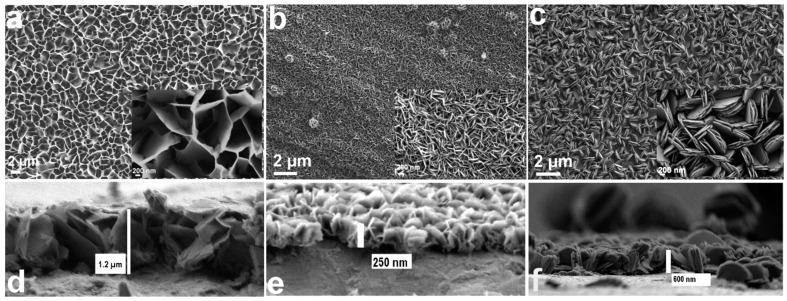
SEM images of the prepared samples: (**a**) standard-procedure β-Ni(OH)_2_ (sample 1), (**b**) sample 2 made with lower ammonia content, and (**c**) sample 3 made only in the presence of nickel chloride hexahydrate and ammonia. The cross sections show (**d**) thin and large-area nanolamellae for sample 1, (**e**) thin but smaller-area nanolamellae for sample 2, and (**f**) stacked disc-like nanolamellae for sample 3.

SEM images of the as-prepared β-Ni(OH)_2_ samples (1, 2, and 3) grown on Ni foil are shown in Figure 3.

Sample 1 (Figure 3a), generated from NiCl_2_.6H_2_O, PEI, and ammonia, has a foam-like morphology composed of thin lamellae that are observed to grow longitudinally (perpendicular to the plane of the substrate). Their lateral dimensions are in the range of few microns, with a height of ~1.2 µm (Figure 3d). Sample 2 (Figure 3b), produced with a relatively lower ammonia content than samples 1 and 3, has, at first glance, a needle-like morphology. However, it is actually composed of similar, albeit smaller, lamellae that are more compact than those in sample 1. Smaller lamellae are observed in the cross-sectional view (Figure 3e), and they exhibit lateral dimensions in the range of several tens of nm, with a height of ~250 nm. Sample 3 (Figure 3c), made only in the presence of NiCl_2_.6H_2_O and ammonia, exhibits a disc-like morphology, with a propensity towards stacking. These lamellae have an average height of ~600 nm and consist of thin layers stacked together (Figure 3f). All samples show lamellae that appear to be tapered, that is, they are thicker at the base and become progressively thinner towards the top. Moreover, the lamellae in sample 1 seem to curl up. These combined features make it very challenging to gauge a sensible estimate of their average thickness.

The longitudinal growth and tapering of the nanolamellae can be explained in terms of both concurrent thermodynamics and kinetics of the growth mechanism. β-Ni(OH)_2_ has a brucite structure; its nucleation and growth are suggested to occur on its lowest energy plane, i.e., the (001) plane [19,36]. The other exposed surface planes of an individual lamella have a relatively higher surface energy, increasing the thermodynamic favorability for the nanolamellae to grow anisotropically, that is, perpendicular to the plane of the substrate, in order to minimize surface energy contribution [37].

(b) Growth on textured Ni substrates

**Figure 4:**
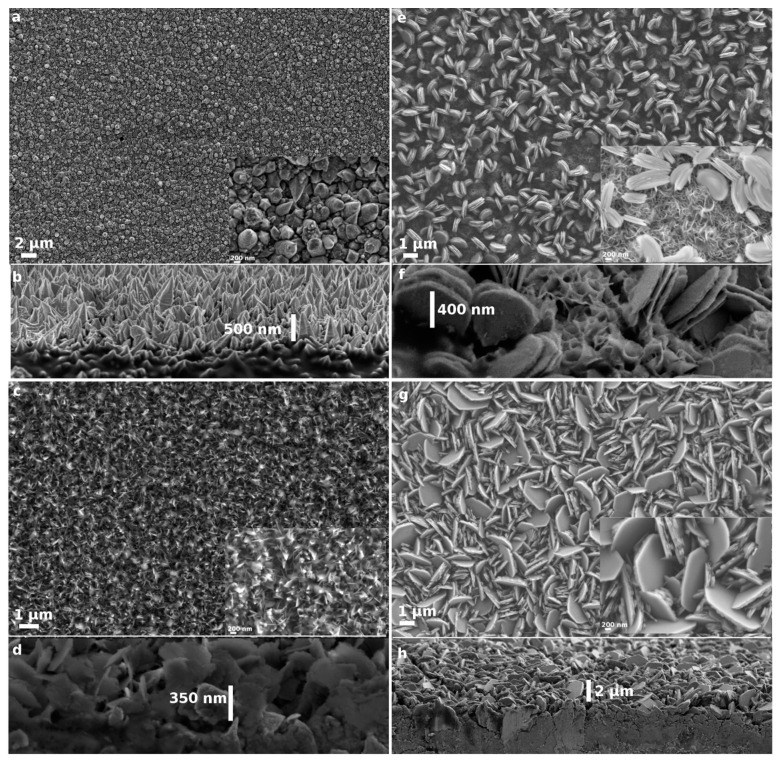
SEM images of the prepared samples: (**a**) Textured nickel substrate composed of nanospikes. (**b**) Cross-sectional SEM showing the nanospikes protruding to about 500 nm above the surface. (**c**) Sample 4 made with the same standard procedure as sample 1 but with a morphology mimicking that of sample 2. (**d**) Cross section of sample 4 showing nanolamellae with a lateral dimension of 350 nm. (**e**) Sample 5 made with lower ammonia content displaying a hierarchal structure; the upper structure comprises disc-like stacked lamellae sitting atop smaller, non-stacked lamellae. (**f**) The upper disc-like structure of sample 5 has a lateral dimension of 400 nm. (**g**) Sample 6 made only in the presence of nickel chloride hexahydrate and ammonia generates large-surface-area lamellae that are stacked. The cross-sectional SEM (**h**) shows that the large lamellae have a lateral dimension of 2 µm.

SEM images of the as-prepared β-Ni(OH)_2_ samples (4, 5, and 6) grown on the textured Ni foil are shown in Figure 4. The textured nickel foil surface shown in Figure 4a has numerous spiky nickel structures protruding up to 500 nm (Figure 4b) from the surface. Each individual spike has a pentagonal base with rough facets that provide access to more nucleating sites than a non-textured surface. Sample 4 (Figure 4c), generated by a similar method as sample 1, has a morphology similar to that of sample 2 (Figure 3b), that is, small lamellae are intertwined between the nickel spikes. Unlike sample 1, we do not observe the formation of large lamellae, despite using a similar concentration of reagents. They exhibit lateral dimensions in the range of several nanometers, with a height of ~350 nm (Figure 4d), which is marginally larger than sample 2 but significantly smaller than sample 1. The presence of more nucleation sites in sample 4 due to its textured nature leads to the formation of more lamellae but of smaller size due to reagent depletion over the course of the reaction. Sample 5 (Figure 4e), which was made using a lower ammonia concentration, produces a surface with two distinct types of morphology. The upper region of the sample is strewn with disc-like structures (similar to sample 3 but with smaller lateral dimension), whereas the bottom is completely covered with a heavily curled-up lamellae structure. The underlying lamella structure is even smaller than that observed in sample 2, despite using a similar concentration. This observation, again, points to the influence of the textured surface, which provides more nucleation sites and causes a reduction in the size of the lamellae. The concentration of ammonia being lower in this case also generates less nickel amine complex that leads to such structure. Whereas over the course of the reaction, the remaining reagents, i.e., nickel chloride and HMTA, react together, the latter decomposes to form ammonia, which leads to subsequently favored growth of the upper-lying disc structure (Figure 4f) that was observed for sample 3 (where only ammonia and nickel chloride were used). Figure 4g,h depict the morphology and a cross-sectional view of sample 6, respectively, when only nickel chloride and ammonia are used on a textured nickel foil. Here, much larger and nearly hexagonally shaped lamellae are formed. They are relatively thick due to the stacking of several lamellae. They are haphazardly angled with respect to each other, most likely due to the slanted angles offered by nickel spikes. Some of those lamellae exhibit lateral dimensions in the range of several micrometers, with a magnitude of ~2 µm (Figure 4h).

### 3.3. Discussion of the Growth Mechanisms

**Figure 5:**
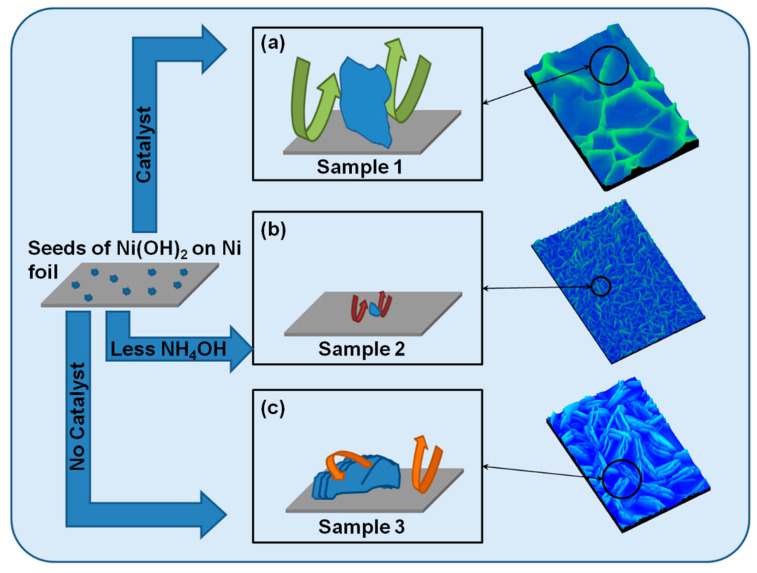
Schematic (not to scale) depicting a possible growth mechanism for (**a**) standard-procedure β-Ni(OH)_2_ (sample 1), (**b**) sample 2 made with lower ammonia content, and (**c**) sample 3 made only in the presence of nickel chloride hexahydrate and ammonia. The larger green arrow indicates catalyzed growth of nanolamellae in (**a**), whereas the smallest red arrows indicate suppressed growth with ammonia as the limiting reagent and (**c**) moderate growth of a stacked morphology when no other additives are present.

The nucleation and growth mechanisms of the of β-Ni(OH)_2_ lamellae are exemplary discussed with reference to the smooth substrate and samples 1–3. A schematic depiction of these mechanisms is rendered in Figure 5.

Kinetically, the steps leading to the formation of β-Ni(OH)_2_ can be explained in the following terms: the combined action of NiCl_2_·6H_2_O and ammonia results in the formation of a deep blue solution containing [Ni(NH_3_)_6_]^2+^_(aq)_. With increasing temperature, the complex decomposes to nickel ions, along with the evolution of ammonia. The latter then supplies the reaction with hydroxyl ions, which combine with Ni ions, leading to nucleation of β-Ni(OH)_2_ on the substrate. The freshly formed β-Ni(OH)_2_ nuclei are attached randomly onto the surface. This is described as the incubation period, during which the newly formed particles are stable in solution. As the reaction proceeds, pH and ammonia concentration decrease. This induces the particles to coalesce with each other to form hierarchical structures. Secondary nucleation can form thicker clusters at the base of the substrate. Anisotropic growth, along with ensuing Ostwald ripening [38], is promoted in the direction of the bulk solution, as the latter has a relatively higher reagent concentration than at the substrate surface. Lateral growth is suppressed when the lamellae come into contact with each other [19]. Tapering of the lamellae occurs as the concentrations of reagents are depleted, thereby terminating the reaction.

In the case of sample 3, since Ni salt and ammonia are the only two reagents, the formation of β-Ni(OH)_2_ is dependent on the rate of decomposition of the amine complex and the rate of uptake of the hydroxyl ions from solution. We suspect that the growth rate is relatively slow with respect to the other samples, as we further elaborate upon shortly. The growth and stacking of the disc-like morphology (Figure 5c) may be a result of the initial formation of thinner lamellae that have unsatisfied bonds on the exposed surfaces with higher surface energy. These provide apt new nucleation sites, which, over time, slowly induce a self-oriented assembly; stacked morphologies eventually form (as seen in Figure 3c) due to an interlayer chemical reaction, which is common within brucite-like structures [39,40].

Various species can be added to the growth solution as either promoters or inhibitors of growth and nucleation of a particular crystal facet, thereby enabling tailoring of the crystal morphology. HMTA is a non-ionic cyclic tertiary amine that is capable of forming a bidentate ligand bridging two metal ions (with oxidation state +2) in solution such that it behaves as a Lewis base [41]. It is also known to decompose to formaldehyde and ammonia under our current reaction conditions [42]. PEI is an additive used to obtain high-aspect-ratio structures, as it promotes longitudinal growth [43]. In the case of sample 1, when HMTA is added to NiCl_2_.6H_2_O, a light blue/green solution is detected; then, when PEI is added, a light purple coloration is observed, indicating that the additives are form complexes with Ni^2+^ in solution. Further addition of ammonia results in a typical deep blue-colored solution, suggesting the overwhelming formation of a Ni amine complex. Sample 1 yielded thinner and much larger individual flakes, as observed in Figure 3a. In this reaction, both the amine complex and HMTA decompose in solution to produce nickel ions, ammonia, and formaldehyde. The decomposition of HMTA provides a constant supply of ammonia, mitigating the pH drop due to the natural evolution of ammonia via evaporation [11], thus behaving as a pH buffer [41]. This excess of ammonia provides an ample supply of hydroxyl ions, which catalyze [9] the formation of β-Ni(OH)_2_, as depicted in Figure 5a.

Large-surface-area lamellae are promoted by PEI, and stacking is limited, as any unsatisfied bonds on the high-energy surfaces are either alleviated by adsorption [9] of un-reacted molecules (HMTA or formaldehyde) or terminated by hydroxyl ions, leading to the formation of thinner lamellae [10,11]. HMTA can also render the lamellae flexible, analogous to other amines [44], which leads to curling at the top edges of the lamellae, especially when the reaction is nearing the termination step. This further assuages any dangling bonds. Sample 2 produces smaller lamellae (Figure 3b) than sample 1, despite the presence of a similar concentration of additives as sample 1. It is reasonable to believe that the lower ammonia content used in this case has a profound effect on the morphology. The lower ammonia content behaves as a limiting agent (as illustrated in Figure 5b), where less nickel amine complex is formed, thereby leading to a smaller structure. Engineering different morphologies is not a trivial matter [11]; thinner lamellae are promulgated to offer better supercapacitive performance [9]. Control of the thickness of the individual lamellae is dependent on the method used to produce them. It has been suggested that the concentration of Ni ions [45] controls the thickness, which correlates well with the lower concentration we used as compared to other literature reports. In our case, we argue that an equivalent concentration of both Ni^2+^ ions and ammonia is required for optimal morphological control of thin lamellae. The rate at which growth occurs can also affect the thickness of the lamellae. In general, the kinetics of the reaction are undoubtedly influenced by additives, temperature, and time of reaction. It has been observed that carrying out such a reaction at a lower temperature (70 °C) results in thicker lamellae with reduced capacitive performance [16]. Thus, the presence of HMTA and a slightly higher temperature (85 °C) promote the growth of thinner lamellae. Decreasing time results in the formation of smaller crystal structures [38] but usually requires higher concentrations of reagents to obtain morphologies with large surface areas. However, we have shown that our CDB method makes use of fewer reagents and takes less time. This enhances its viability to reduce cost and to control the morphology of β-Ni(OH)_2_ while attempting to attain improved capacitive performance.

### 3.4. Electrochemical Characterization of the Electrodes

**Figure 6:**
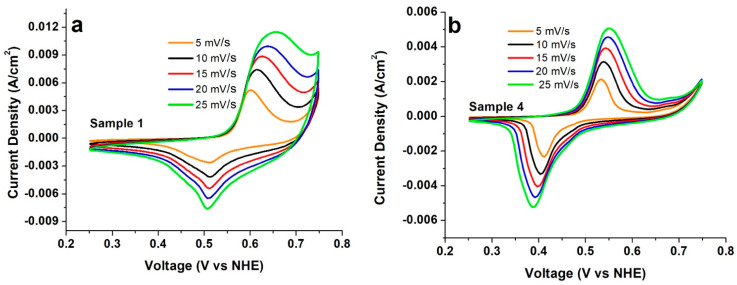
Exemplary CV curves collected at different scan rates for sample 1 (**a**) and sample 4 (**b**); sample 1 was prepared following the standard procedure. Sample was 4 prepared using a similar standard procedure as that for sample 1 but on a textured substrate. The CV curves of the other samples are shown comparatively in Figure S1 (Supplementary Materials).

In this study, cyclic voltammetry (CV) is used to potentiostatically determine the electrochemical properties of β-Ni(OH)_2_ film electrodes. In general, two strong peaks (See Figure 6a,b and Figure S1a–d) are observed in each curve, which correspond to the faradaic oxidation/reduction reactions. It is interesting to note that sample 4 is characterized by rather sharp oxidation and reduction peaks that are more akin to “battery-like” behavior. This might be amenable to the very fine lamellae nanostructure obtained for this sample on the textured Ni substrate. As discussed later with respect to the galvanic charging–discharging studies, one might attribute both a pseudocapacitor and battery-like character to this sample.

**Figure 7:**
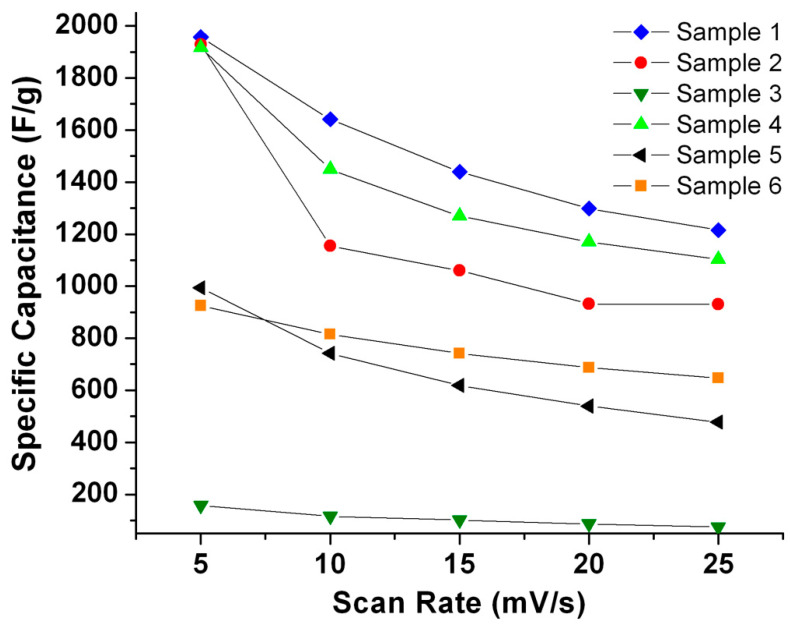
The specific capacitance vs. the scan rate of the different samples calculated from the potentiostatic data (Equation (2)).

Figure 7 shows the specific capacitance of the samples calculated from the cyclovoltammograms at different scan rates in 1 M KOH. The scan rates were, 5, 10, 15, 20, and 25 mV s^−1^. The faradaic reaction proceeds according to the mechanism expressed by Reaction (1):

Ni(OH)_2_ + OH^−^ ↔ NiOOH + H_2_O + e^−^ (1)

Reaction (1) is diffusion-limited, as it depends on the rate at which the hydroxyl ions interact with the electrode at its interface [2,9]. In all cases, as the scan rate is increased, the potential of the oxidation peak and the potential of the reduction peak shift towards more positive and negative values, respectively. The current densities recorded for all six samples are also observed to increase with increasing scan rates. Focusing on the oxidation peaks, higher current densities are recorded for samples 1 and 6, whereas sample 5 has the lowest current density, with intermediary current densities are noted for the other samples. At higher scan rates, the rate of ion/electron diffusion is relatively faster, thereby limiting the participation of inner active sites; less interfacial material and faster ion/electron mobility contribute to the lowering of the overall resistivity of the surface, giving rise to higher current densities [2,9]. Therefore, a lower scan rate is preferable to promote slow diffusion of hydroxyl ions, enabling all active sites to sustain the redox reaction. The latter provides a better gauge of the specific capacitance of the surface under potentiostatic conditions.

The specific capacitance of the samples at different scan rates was determined from the CV curves using the following equation:

(2) C=12·∆V·m∫VinitialVfinal│I│dVdt·dV

where *C* is the specific capacitance, *V_initial*/*final_* is the starting/end potential for one cycle, Δ*V* is the voltage range (*V_final_*–*V_initial_*), *m* is the mass of the electrode material, │*I*│ is the instantaneous current at a given potential, and (*dV*/*dt*) is the scan rate. A graph of the specific capacitance vs. scan rate for all six samples is shown in Figure 7. All samples show a decrease in specific capacitance with an increase in scan rate (Figure 7), as the specific capacitance is inversely proportional to the scan rate. It should be noted that the behavior of sample 4 is in line with that of the other samples, which also stresses its pseudocapacitor character. 

The values of the specific capacitance computed at a scan rate of 5 mVs^−1^ were found to be 1957, 1928, 158, 1916, 994, and 925 Fg^−1^ for samples 1 to 6, respectively (Figure 7). The large span of these values underlines the drastic impact of structural morphologies on electrochemical performance. Under the current potentiostatic conditions, sample 1, with large-surface-area lamellae, shows the highest specific capacitance. The thin lamellae are irregularly interconnected, forming macropores (Figure 3a). This promotes more accessibility for the electrolyte, which can interact more efficiently with the large surface area during the charge transfer reaction. Moreover, the thin lamella structure decreases the ion diffusion pathway within the active material. Furthermore, volume expansion or electrode swelling [46,47,48] is known to result from ions diffusing within β-Ni(OH)_2_. Thus, more interspatial voids enhance the expansion, which is the case in sample 1. In contrast, stacking, which is present in sample 3, is deemed to inhibit such swelling, curtailing the full use of all active sites. Sample 2 offers thin lamellae, albeit with smaller area, which gives rise to a specific capacitance of 1928 Fg^−1^ at a scan rate 5 mVs^−1^; however, a rapid decrease to 930 Fg^−1^ at a scan rate of 25 mVs^−1^ entails a 48% specific capacity retention. Sample 3 provides neither the highest specific capacitance nor the best specific capacity retention; the latter drops to a mere 46% when the scan rate is increased from 5 to 25 mVs^−1^. Sample 1 preserves a 62% specific capacity retention (from 1957 to 1215 Fg^−1^ at 5 and 25 mVs^−1^, respectively) under potentiostatic conditions. Sample 4 retains 58% specific capacity when the scan rate is changed from 5 to 25 mVs^−1^ from an initial specific capacitance of 1916 Fg^−1^ at 5 mVs^−1^. The latter value is very close to that of sample 2, with which it shares similitude in terms of morphology. However, the textured substrate of sample 4 imparts it with what seems to be a better stability with increasing scan rate. Samples 5 and 6 have intermediary specific capacitances of 993 and 925 Fg^−1^ at 5 mVs^−1^, respectively. Under potentiostatic conditions, the specific capacity retention correspondingly drops to 48% and 70% when the scan rate is increased to 25 mVs^−1^. Sample 4 has a better specific capacitance than sample 3, with which it shares similar topographic morphology, due to the nature of the textured surface of the substrate. Similarly, the large surface area of the lamellae present in sample 6 (Figure 4g) affords for a better capacitance when compared to sample 3. However, the specific capacitance of both samples 4 and 5 is much lower than that of samples 1, 2, and 4 due to stacking of the lamellae. As pointed out earlier, this lies in the stacking morphology, which inhibits full use of all active sites.

**Figure 8:**
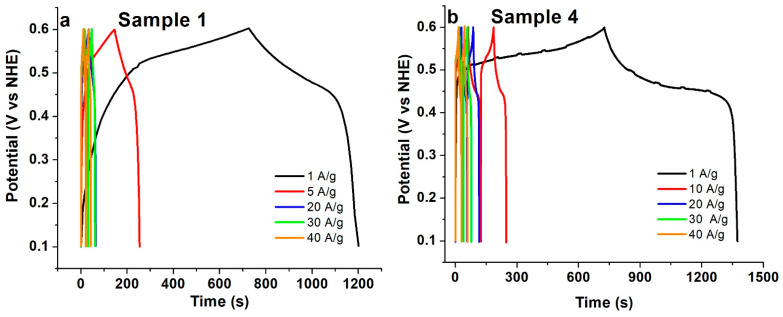
Charge–discharge curves collected at different current densities for (**a**) sample 1 (standard-procedure β-Ni(OH)_2_ on a flat Ni substrate) and (**b**) sample 4 (similar procedure but on a textured Ni substrate). The charge–discharge curves of the other samples are shown in Figure S2 (Supplementary Materials). The small “bumps” in the low-rate charging–discharging curve of sample 4 are probably due to measurement artifacts.

Under potentiostatic conditions, the voltage window and scan rate (*dV*/*dt*) are fixed, while the variation in current density is monitored. However, a better gauge of super capacitive performance is achieved using galvanostatic charging/discharging. This enables the use of various constant current densities to be supplied to the electrode under a stable potential window while the time taken for both the charge and discharge cycle(s) is monitored. All samples present profiles for charging and discharging that are non-linear, confirming the pseudocapacitive nature of the electrodes (See Figure 8 and Figure S2) (a double-layer capacitor ideally affords a triangular-wave-like profile for a charge–discharge cycle). The specific capacitance is calculated from the constant current discharge curve using Equation (3):

(3) $$C=\frac{I\cdot ∆t}{m\cdot ∆V}$$

where *C* is the specific capacitance, *m* is the mass of the electrode material, *I* is the discharge current, Δ*V* is the potential window, and Δ*t* is the time taken to discharge the supercapacitor.

**Figure 9:**
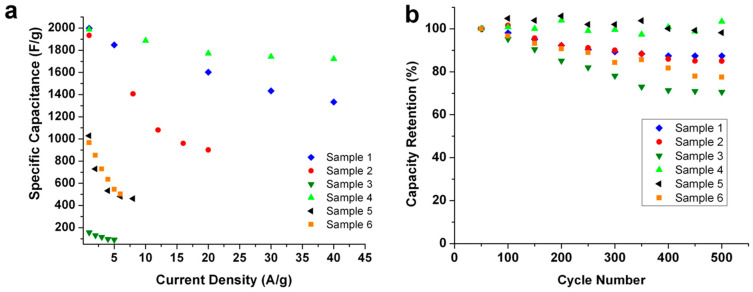
(**a**) Specific capacitance calculated based on galvanostatic discharge cycle(s) at different current densities for all six samples. (**b**) The capacity retention properties for samples 1 to 6 monitored using current densities of 30, 20, 3, 40, 6, and 6 Ag^−1^, respectively.

Once more, the behavior of sample 4 differs from that of sample 1, exhibiting plateau-like charging–discharging curves over a long span of time. As mentioned above, this could be ascribed to a “battery-like” character; its high retention rate observed in Figure 9a (see below) further supports the mixed character of this sample. Similar behavior was observed by Long et al. on carbon nanofoam MnO_x_ nanocomposite electrodes [49]. The values of specific capacitance calculated from galvanostatic discharge measurements at low current density are marginally higher than the values computed from the potentiostatic measurements. This is consistent with literature reports and is ascribed to the slow and more efficient diffusion of ions/electrons through the interfacial surface of the electrode during the charge transfer process [2]. However, the propensity of a capacitor to be discharged at a high rate would be advantageous for its commercial implementation. Under galvanostatic conditions, that is, constant current density, both sample 1 and sample 4 have the highest specific capacitance; both samples have thin individual lamellae that can sustain higher current densities (Figure 9a) while maintaining their redox behavior. The large surface area of the individual lamellae in sample 1 can explain its better performance, despite the smaller surface area of the individual lamella; sample 4 has a better specific capacitance than sample 1. This is due to the nature of the textured substrate providing a better conducting pathway. Sample 2 corroborates this finding, as it has a similar morphology to that of sample 4, but the absence of a textured substrate surface limits both its specific capacitance and redox behavior at higher current densities (Figure 9a). Other samples, such as sample 3, 5, and 6, with thick individual lamellae that are also stacked, have low specific capacitance and are not able to sustain large current density. Nevertheless, sample 5 and 6 have a relatively higher specific capacitance than sample 3 due to the textured surface of the substrate (Figure 9a). As mentioned before, the pseudocapacitance is diffusion-limited and depends on the rate at which the electrolyte ions interact with the electrode [2,9]. Samples with stacked morphology afford limited access to all sites; they cannot sustain the redox reaction at higher current densities, making them less suitable for commercial use. Consequently, according to our galvanostatic experiment, electrodes with thin and large-area lamellae grown in the presence of a textured substrate have enhanced specific capacitance.

Another important characteristic of an electrochemical capacitor is its capacitive performance. This is measured in terms of capacity retention, which is usually recorded over a large number of cycles. The cycling capability (or cycling life) of an electrode provides a measure of its long-term cycling performance (also termed capacitive performance). Figure 9b shows the capacity retention for samples 1 to 6 over 500 cycles using current densities of 30, 20, 3, 40, 6, and 6 Ag^−1^, respectively. The capacitive retention of samples 1 to 6 is 87, 84, 71, 100, 98, and 77%, respectively. Samples prepared on a textured substrate show better capacitive performance than their counterparts prepared on flat substrates. In general and as already mentioned, samples with relatively thicker lamellae in stacked morphology are less promising. Sample 5, which has a hierarchical structure (Figure 4e) consisting of two distinct morphologies, has a capacitive performance that is observed to first increase with increasing cycle number (107% at 200 cycles), then decrease. The specific capacitance of sample 5 is only 482 Fg^−1^ at 6 Ag^−1^, and such an increase is commonly associated with incomplete conditioning of the sample prior to measurement. Only with time do both morphologies slowly begin to contribute to the capacitive performance until it stabilizes. Sample 4 has the best capacitive performance, as it retains 100% of its initial specific capacitance when discharged at a higher current density (40 Ag^−1^) than the other samples. The values are observed to fluctuate within 100 ± 3% under experimental conditions over the course of 500 cycles. The enhanced specific capacitance recorded for sample 4 (e.g., 1721 Fg^−1^ at 40 Ag^−1^) is vastly superior to previously reported data using CBD [15] (1098 Fg^−1^ at 40 Ag^−1^) and is better than that reported for similar material made using the hydrothermal method [12] (1778 Fg^−1^ at 2.5 Ag^−1^). The specific capacitance obtained using our relatively fast and cost-effective CBD method is also superior to that of NiO nanorods obtained via Ni electrodeposition and oxidation in porous anodic alumina but is inferior to that of NiO nanotubes produced using the same processing method [50]. We strongly believe that further improvements can be achieved by making use of asymmetrical electrochemical capacitors, which can expand the current 0.5 V operating potential window by making use of counter electrodes made of carbon allotropes. Table 2 summarizes our results and compares them to some of the best results reported in the literature using similar processing methods.

## 4. Conclusions

Tailoring the process conditions of chemical bath deposition allows various morphologies of β-Ni(OH)_2_ to be deposited on Ni substrates. Different morphologies were synthesized not only to understand their influence on supercapacitive behavior but also to gather further insights as to how the concentration and presence of different reagents/catalysts alters the resulting morphology. We found that nanolamellae formation is dependent on an equivalent amount of Ni^2+^ and ammonia, while additives such as HMTA and PEI promote the formation of thin nanolamellae with large surface areas. The use of a textured substrate is not only observed to alter the morphology of the active material but also enhances the specific capacitance and capacitive performance of the electrode. Stacked β-Ni(OH)_2_ lamellae afford low-specific-capacitance as ionic diffusion is limited in this such structures, whereas nanolamellae with larger surface areas have better specific capacitance. We have shown that β-Ni(OH)_2_ electrode films made under our CBD conditions offer a better specific capacitance (1961 Fg^−1^ at 5 mVs^−1^ and 1998 Fg^−1^ at 1 Ag^−1^), with excellent capacity retention during cycling at 40 Ag^−1^. CBD is a cost-effective and versatile method apt for large-scale implementation, as the electrode is grown directly on a substrate over less time compared to hydrothermal methods. This makes the use of a binder superfluous, while delivering capacitance results that are advantageously comparable to those published in the literature. Moreover, our electrodes are made using very low reagent concentrations, yet still yield enhanced performance when compared to previous literature data (see Table 2). This provides an elegant method to resolve issues of poor cycling performance associated with certain electrodes made of similar materials. Our economical method provides a platform for future implementation of such electrodes as energy storage devices with the aim of harnessing green energy vectors.

## Figures and Tables

**Table 1:** Summary of the reagent compositions and preparation conditions of the samples.

	Synthesis Procedure	Sample #
Smooth Ni substrate	40 mM NiCl_2_·6H2O + 50 mM HMTA, dissolution in DI water by stirring + drop-wise addition of 75 µL PEI and 6 mL of 25% NH_4_OH; stirring; chemical bath maintained at 85–88 °C for 2 h	Sample 1
	Same reagents and conditions as above but less NH_4_OH (0.6 mL)	Sample 2
	Same basic reagents and conditions as for sample 1 but without the addition of HMTA and PEI	Sample 3
Textured Ni substrate (see Section 2.2 for the electrochemical preparation of the textured Ni substrate)	Same conditions as sample 1	Sample 4
	Same conditions as sample 2	Sample 5
	Same conditions as sample 3	Sample 6

**Table 2:** Summary of the best results obtained in the present work in comparison to some of the best results published in the literature using CBD or hydrothermal methods.

Crystal Variant	Processing Route	Capacitance, F/g	Reference	Remarks
β-Ni(OH)_2_	CBD	1721 at 40 A/g and 1998 at 1 A/g	This work	Textured Ni
β-Ni(OH)_2_	Hydrothermal	1778 at 2.5 A/g	[12]	On Ni foam
β-Ni(OH)_2_	CBD	1098 at 40 A/g	[15]	On Ni foam
β-Ni(OH)_2_	Hydrothermal	1470 at 2 A/g	[24]	On Ni foam/binder
α-Ni_x_Co_(1−x)_(OH)_2_	Hydrothermal	1396 at 20 A/g	[25]	Contains intercalated BO_2_^−^ anions on rGO
β-Ni(OH)_2_	KOH etching of layered NiAl hydroxide	1471 at 2.5 A/g	[26]	On Ni foam/binder
Ni(OH)_2_/Au-NPs	Hydrothermal	1927 at 1 A/g	[27]	On Ni foam/binder

## Data Availability

The data presented in this study are available on request from the corresponding author.

## Supplementary Materials

The following supporting information can be downloaded at: https://www.mdpi.com/article/10.3390/mi14081644/s1, Figure S1: The cyclovoltammograms (potentiostatic condition) of the samples under various scan rates, Figure S2: The charge–discharge behavior (galvanostatic conditions) of the samples under various current densities.

## References

[1] Hu C.-C., Chang K.-H., Lin M.-C., Wu Y.-T. (2006). Design and Tailoring of the Nanotubular Arrayed Architecture of Hydrous RuO_2_ for Next Generation Supercapacitors. Nano Lett..

[2] Liang K., Tang X., Hu W. (2012). High-performance three-dimensional nanoporous NiO film as a supercapacitor electrode. J. Mater. Chem..

[3] Yang G.W., Xu C.L., Li H.L. (2008). Electrodeposited nickel hydroxide on nickel foam with ultrahigh capacitance. Chem. Commun..

[4] Wadley H.N.G. (2002). Cellular Metals Manufacturing. Adv. Eng. Mater..

[5] Zhao D., Zhou W., Li H. (2007). Effects of Deposition Potential and Anneal Temperature on the Hexagonal Nanoporous Nickel Hydroxide Films. Chem. Mater..

[6] Wang X., Wang Y., Zhao C., Zhao Y., Yan B., Zheng W. (2012). Electrodeposited Ni(OH)_2_ nanoflakes on graphite nanosheets prepared by plasma-enhanced chemical vapor deposition for supercapacitor electrode. New J. Chem..

[7] Sharma P.K., Fischer H., Craievich A.F. (1999). Chemical and Structural Properties of Nickel Hydroxide Xerogels Obtained by the Sol-Gel Procedure in the Presence of Acetic Acid. J. Am. Ceram. Soc..

[8] Lin C., Al-Muhtaseb S.A., Ritter J.A. (2003). Thermal Treatment of Sol-Gel Derived Nickel Oxide Xerogels. J. Sol-Gel Sci. Technol..

[9] Jiang H., Zhao T., Li C., Ma J. (2011). Hierarchical self-assembly of ultrathin nickel hydroxide nanoflakes for high-performance supercapacitors. J. Mater. Chem..

[10] Yang D., Wang R., He M., Zhang J., Liu Z. (2005). Ribbon- and Boardlike Nanostructures of Nickel Hydroxide: Synthesis, Characterization, and Electrochemical Properties. J. Phys. Chem. B.

[11] Buscaglia M.T., Buscaglia V., Bottino C., Viviani M., Fournier R., Sennour M., Presto S., Marazza R., Nanni P. (2008). Morphological Control of Hydrothermal Ni(OH)_2_ in the Presence of Polymers and Surfactants: Nanocrystals, Mesocrystals, and Superstructures. Cryst. Growth Des..

[12] Suo S.L.H., Wang J., Wang Y., Zhao C., Xing S. (2012). Facile synthesis of nanostructured Ni(OH)_2_ on nickel foam and its electrochemical property. Colloids Surf. Physicochem. Eng. Asp..

[13] Gund G.S., Dubal D.P., Shinde S.S., Lokhande C.D. (2013). One step hydrothermal synthesis of micro-belts like β-Ni(OH)_2_ thin films for supercapacitors. Ceram. Int..

[14] Patil U.M., Gurav K.V., Fulari V.J., Lokhande C.D., Joo O.S. (2009). Characterization of honeycomb-like “β-Ni(OH)_2_” thin films synthesized by chemical bath deposition method and their supercapacitor application. J. Power Sources.

[15] Yuan Y.F., Xia X.H., Wu J.B., Yang J.L., Chen Y.B., Guo S.Y. (2011). Nickel foam-supported porous Ni(OH)_2_/NiOOH composite film as advanced pseudocapacitor material. Electrochim. Acta.

[16] Dubal D.P., Fulari V.J., Lokhande C.D. (2012). Effect of morphology on supercapacitive properties of chemically grown β-Ni(OH)_2_ thin films. Microporous Mesoporous Mater..

[17] Schwartz R.W., Schneller T., Waser R. (2004). Chemical solution deposition of electronic oxide films. Comptes Rendus Chim..

[18] Matsui K., Kyotani T., Tomita A. (2002). Hydrothermal Synthesis of Single-Crystal Ni(OH)_2_ Nanorods in a Carbon-Coated Anodic Alumina Film. Adv. Mater..

[19] Luo Y., Duan G., Li G. (2007). Synthesis and characterization of flower-like β-Ni(OH)_2_ nanoarchitectures. J. Solid State Chem..

[20] Zhu Z., Wei N., Liu H., He Z. (2011). Microwave-assisted hydrothermal synthesis of Ni(OH)_2_ architectures and their in situ thermal convention to NiO. Adv. Powder Technol..

[21] Xiao T., Hu X., Heng B., Chen X., Huang W., Tao W., Wang H., Tang Y., Tan X., Huang X. (2013). Ni(OH)_2_ nanosheets grown on graphene-coated nickel foam for high-performance pseudocapacitors. J. Alloys Compd..

[22] Aricò A.S., Bruce P., Scrosati B., Tarascon J.M., van Schalkwijk W. (2005). Nanostructured materials for advanced energy conversion and storage devices. Nat. Mater..

[23] Simon P., Gogotsi Y. (2008). Materials for electrochemical capacitors. Nat. Mater..

[24] Liu Y., Liu N., Hu J., Xu C., Wang S., Du G. (2020). In situ growth of Ni(OH)_2_ nanoflakes on Ni foam as binder- free electrode for electrochemical pseudocapacitor. IOP Conf. Ser. Earth Environ. Sci..

[25] Xin Y., Dai X., Lv G., Wei X., Li S., Li Z., Xue T., Shi M., Zou K., Chen Y. (2021). Stability-Enhanced α-Ni(OH)_2_ Pillared by Metaborate Anions for Pseudocapacitors. ACS Appl. Mater. Interfaces.

[26] Chen L., Yang X., Tian Y., Wang Y., Zhao X., Lei X., Zhang F. (2022). Fabrication of β-Ni(OH)_2_ Particles by Alkaline Etching Layered Double Hydroxides Precursor for Supercapacitor. Front. Energy Res..

[27] Kim S.-I., Thiyagarajan P., Jang J.-H. (2014). Great improvement in pseudocapacitor properties of nickel hydroxide via simple gold deposition. Nanoscale.

[28] Li M., Hu A., Tao Y. (2013). Superhydrophobicity of different shaped Ni surfaces fabricated with electrodeposition. Surf. Innov..

[29] Bode H., Dehmelt K., Witte J. (1966). Zur kenntnis der nickelhydroxidelektrode—I. Über das nickel (II)-hydroxidhydrat. Electrochim. Acta.

[30] Hall D.S., Lockwood D.J., Poirier S., Bock C., MacDougall B.R. (2012). Raman and Infrared Spectroscopy of α and β Phases of Thin Nickel Hydroxide Films Electrochemically Formed on Nickel. J. Phys. Chem. A.

[31] Ramesh T.N., Kamath P.V., Shivakumara C. (2005). Correlation of Structural Disorder with the Reversible Discharge Capacity of Nickel Hydroxide Electrode. J. Electrochem. Soc..

[32] Langford J.I., Wilson A.J.C. (1978). Scherrer after sixty years: A survey and some new results in the determination of crystallite size. J. Appl. Cryst..

[33] Cairns R.W., Ott E. (1933). X-Ray Studies of the System Nickel—Oxygen—Water. I. Nickelous Oxide and Hydroxide. J. Am. Chem. Soc..

[34] Bantignies J.L., Deabate S., Righi A., Rols S., Hermet P., Sauvajol J.L., Henn F. (2008). New Insight into the Vibrational Behavior of Nickel Hydroxide and Oxyhydroxide Using Inelastic Neutron Scattering, Far/Mid-Infrared and Raman Spectroscopies. J. Phys. Chem. C.

[35] Bernard M.C., Cortes R., Keddam M., Takenouti H., Bernard P., Senyarich S. (1996). Structural defects and electrochemical reactivity of β-Ni(OH)_2_. J. Power Sources.

[36] Wang Y., Gai S., Li C., He F., Zhang M., Yan Y., Yang P. (2013). Controlled synthesis and enhanced supercapacitor performance of uniform pompon-like β-Ni(OH)_2_ hollow microspheres. Electrochim. Acta.

[37] Yang L.X., Zhu Y.J., Tong H., Liang Z.H., Wang W.W. (2007). Hierarchical β-Ni(OH)_2_ and NiO Carnations Assembled from Nanosheet Building Blocks. Cryst. Growth Des..

[38] Yuan C., Zhang X., Su L., Gao B., Shen L. (2009). Facile synthesis and self-assembly of hierarchical porous NiO nano/micro spherical superstructures for high performance supercapacitors. J. Mater. Chem..

[39] Benito P., Labajos F.M., Rives V. (2006). Microwave-treated layered double hydroxides containing Ni^2+^ and Al^3+^: The effect of added Zn^2+^. J. Solid State Chem..

[40] Carpani I., Giorgetti M., Berrettoni M., Buldini P.L., Gazzano M., Tonelli D. (2006). A new approach for the synthesis of K^+^-free nickel hexacyanoferrate. J. Solid State Chem..

[41] Greene L.E., Yuhas B.D., Law M., Zitoun D., Yang P. (2006). Solution-Grown Zinc Oxide Nanowires. Inorg. Chem..

[42] Strom J.G., Jun H.M. (1980). Kinetics of hydrolysis of methenamine. J. Pharm. Sci..

[43] Moonoosawmy K.R., Es-Souni M., Minch R., Dietze M., Es-Souni M. (2011). Template-assisted generation of three-dimensionally branched titania nanotubes on a substrate. Cryst. Eng. Comm..

[44] Sampanthar J.T., Zeng H.C. (2002). Arresting Butterfly-Like Intermediate Nanocrystals of β-Co(OH)_2_ via Ethylenediamine-Mediated Synthesis. J. Am. Chem. Soc..

[45] Li Y., Tan B., Wu Y. (2008). Ammonia-Evaporation-Induced Synthetic Method for Metal (Cu, Zn, Cd, Ni) Hydroxide/Oxide Nanostructures. Chem. Mater..

[46] Wang J., Pang H., Yin Y., Guan L., Lu Q., Gao F. (2010). Controlled fabrication and property studies of nickel hydroxide and nickel oxide nanostructures. CrystEngComm.

[47] Ida S., Shiga D., Koinuma M., Matsumoto Y. (2008). Synthesis of Hexagonal Nickel Hydroxide Nanosheets by Exfoliation of Layered Nickel Hydroxide Intercalated with Dodecyl Sulfate Ions. J. Am. Chem. Soc..

[48] Li W., Zhang S., Chen J. (2005). Synthesis, Characterization, and Electrochemical Application of Ca(OH)_2_-, Co(OH)_2_-, and Y(OH)_3_-Coated Ni(OH)_2_ Tubes. J. Phys. Chem. B.

[49] Long J.W., Sassin M.B., Fischer A.E., Rolison D.R., Mansour A.N., Johnson V.S., Stallworth P.E., Greenbaum S.G. (2009). Multifunctional MnO_2_-Carbon Nanoarchitectures Exhibit Battery and Capacitor Characteristics in Alkaline Electrolytes. J. Phys. Chem. C.

[50] Dar F.I., Moonooswamy K.R., Es-Souni M. (2013). Morphology and property control of NiO nanostructures for supercapacitor applications. Nanoscale Res. Lett..

