# Supplementary material for: Towards High Capacitive Performance of Chemically Deposited β-Ni(OH)2 Nanolamellae Electrode Films
Source: Micromachines (Basel). 2023 Aug 20;14(8):1644. doi: 10.3390/mi14081644 (PMC10456634; doi:10.3390/mi14081644)
Supplement: Supplementary file 1 [file micromachines-14-01644-s001.zip › micromachines-2538891-supplementary.pdf]

Supporting Information for

Towards High Capacitive Performance of Chemically Deposited β-$Ni(OH)_2$ Nanolamellae Electrode Films

Kevin Radakishna Moonooswamy[1], and Mohammed Es-Souni [2] *

[1]Currently with Toronto Metropolitan University, Victoria Street 350, Ontario, Canada

[2] Formerly with Kiel University of Applied Sciences, Grenzstrasse 3, Kiel 24149, Germany.

* **Correspondence:** mohammed.es-souni@fh-kiel.de

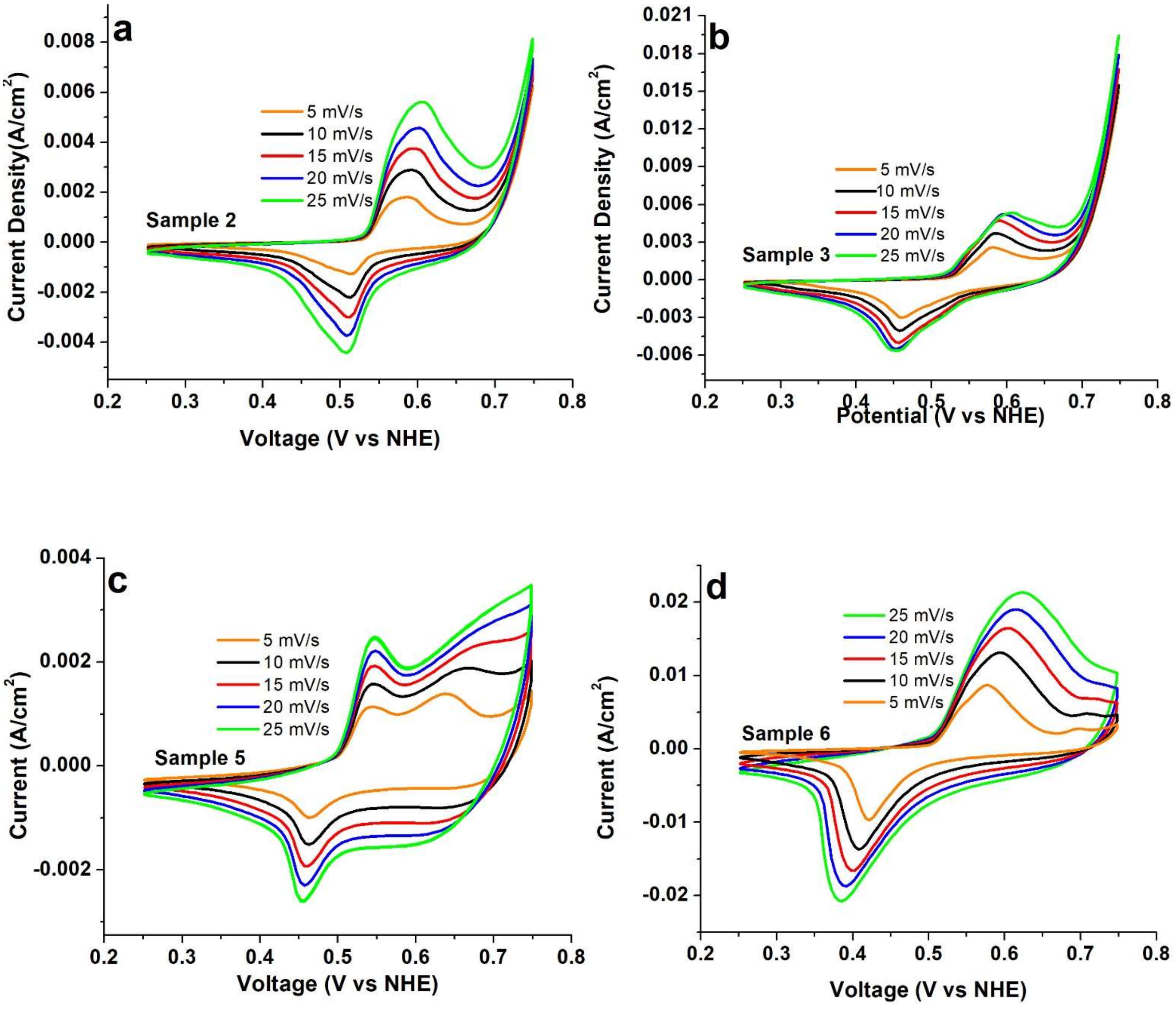

Figure S1. CV curves collected at different scan rates for (a) sample 2 made with lower ammonia content, (b) sample 3 made only in the presence of nickel chloride hexahydrate and ammonia, (c) sample 5 made with lower ammonia content similar to sample 2 but on a textured substrate and (d) sample 6 made only in the presence of nickel chloride hexahydrate and ammonia similar to sample 3 but on a textured substrate. The double peaks observed at lower scan rates in (c) is due to incomplete conditioning of the hierarchal structure at 25 mVs^{-1}.

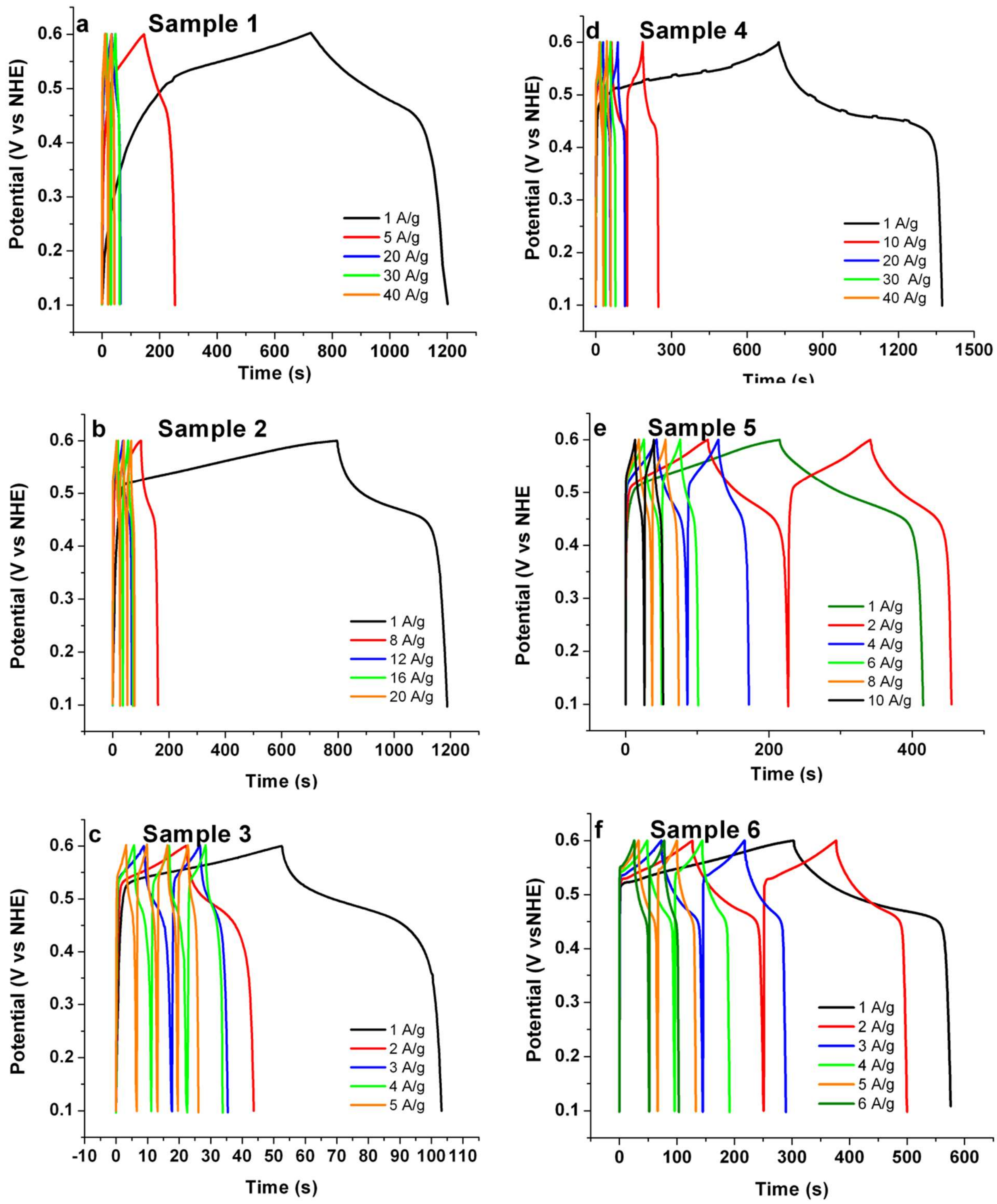

Figure S2. Charge/Discharge curves collected at different current density for (a) standard procedure β-$Ni(OH)_2$ sample 1, (b) sample 2 made with lower ammonia content, (c) sample 3 made only in the presence of nickel chloride hexahydrate and ammonia, (d) sample 4 made with similar standard procedure as sample 1 but on a textured substrate, (e) sample 5 made with lower ammonia content similar to sample 2 but on a textured substrate and (f) sample 6 made only in

the presence of nickel chloride hexahydrate and ammonia similar to sample 3 but on a textured substrate.
